# Impact of frailty and its change on urinary incontinence: A longitudinal analysis from two prospective study of ageing

**DOI:** 10.1371/journal.pone.0330062

**Published:** 2025-08-20

**Authors:** Xiaohan Gan, Linghao Meng, Shangqi Cao, Hexiang Bai, Xiang Li

**Affiliations:** 1 West China Hospital, West China Medical School, Sichuan University, Chengdu, China; 2 Department of Urology, Institute of Urology, West China Hospital, West China Medical School, Sichuan University, Chengdu, China; National Trauma Research Institute, AUSTRALIA

## Abstract

**Background:**

This study aimed to longitudinally investigate the impact of baseline frailty status and its changes over time on the risk of incident urinary incontinence (UI) in older adults, using data from two large prospective cohorts.

**Methods:**

This study included community-dwelling older participants from China Health and Retirement Longitudinal Study (CHARLS) and Health and Retirement Study (HRS). Frailty status was assessed using a Frailty Index (FI) categorized as robust, pre-frail, and frail. Changes in frailty status were evaluated at baseline and at a second survey two years later. The primary outcome was incident UI, determined by participant self-report. Cox proportional hazard regression models were used to calculate Hazard Ratios (HRs) and 95% Confidence Intervals (CIs), adjusting for multiple covariates.

**Results:**

In the baseline frailty status analysis, a total of 12,398 participants from CHARLS and 13,817 from HRS were included. Compared to robust participants, frail participants had a significantly increased risk of incident UI (CHARLS: HR 4.51, 95% CI 3.77–5.38; HRS: HR 2.25, 95% CI 2.07–2.46), and pre-frail participants also had a significantly increased risk (CHARLS: HR 2.14, 95% CI 1.79–2.55; HRS: HR 1.50, 95% CI 1.38–1.62). In the analysis of changes in frailty status, participants who progressed from robust to pre-frail/frail had a significantly increased risk of incident UI compared to those who remained robust (CHARLS: HR 2.81, 95% CI 1.94–4.08; HRS: HR 1.44, 95% CI 1.24−1.68). Conversely, participants who recovered from frail to robust/pre-frail had a significantly decreased risk of incident UI compared to those who remained frail(CHARLS: HR 0.51, 95% CI 0.41–0.62; HRS: HR 0.79, 95% CI 0.69–0.90). Subgroup analyses revealed similar results.

**Conclusion:**

Frailty and worsening frailty status are significantly associated with an increased risk of Urinary Incontinence in older adults, while recovery of frailty status is associated with a decreased risk.

## Introduction

Urinary incontinence (UI), defined as any involuntary leakage of urine, is a significant health issue, particularly in the rapidly growing elderly population [1]. Studies in community-dwelling populations report prevalence rates ranging from 7% to as high as 42% [2,3]. It is crucial to recognize that UI is not an inevitable consequence of aging but a significant health problem requiring attention and intervention [4]. The multiple and profound negative impacts of UI across physical, psychological, and social domains highlight the critical importance of identifying its risk factors and developing effective strategies for prevention, early detection, and comprehensive management to reduce its harmful effects on the well-being of older adults and their caregivers.

Frailty is recognized as a key syndrome in geriatric medicine, characterized by increased vulnerability to stressors due to age-related decline in physiological reserves and function across multiple organ systems [5,6]. Frail older adults are more susceptible to a range of adverse health outcomes, including falls, disability, hospitalization, and mortality [7]. This makes identifying and managing frailty an important focus in geriatric research and clinical practice. Recognizing frailty enables proactive interventions that can potentially prevent or delay the occurrence of these adverse outcomes. Frailty can be assessed using various validated tools and methods [8]. Among these, the Frailty Index (FI) is based on the concept of cumulative deficits, quantifying frailty by calculating the number of health-related deficits an individual has accumulated relative to the total number of deficits considered [5]. These deficits can cover a wide range of factors, including diseases, disabilities, symptoms, and abnormal laboratory results [9,10].

Increasing evidence from epidemiological and longitudinal studies suggests a significant and complex relationship between frailty and UI in older adults [3,11]. While existing studies have established the association between frailty and UI, there is a relative scarcity of longitudinal studies specifically designed to examine the impact of changes in individual frailty status over time on the risk of UI onset or remission, particularly those utilizing the wealth of data from large national databases. This study directly addresses a key gap in the current literature by prospectively investigating the longitudinal relationship between baseline frailty levels and changes in frailty status over time with the risk of UI in large populations from China and the United States, utilizing data from the China Health and Retirement Longitudinal Study (CHARLS) and the Health and Retirement Study (HRS) databases.

## Methods

### Study design and participants

This study used data from two large, ongoing health studies: the CHARLS in China and the HRS in the USA [12,13]. Both are long-term studies that track the health of a large group of people across the country. For this research, we used information from the 2011 survey (Wave 1) of CHARLS and the 2006 survey (Wave 8) of HRS as the baseline. We also used data from the 2013 CHARLS survey (Wave 2) and the 2008 HRS survey (Wave 9) as the second check-in to see how frailty status changed over time. We then followed participants through later surveys until 2020 (Wave 5 for CHARLS and Wave 16 for HRS) to observe the main outcome of interest. The Peking University Ethical Review Committee gave the CHARLS their seal of approval (IRB00001052–11015). At the time of participation, each respondent signed the informed consent form, and they were assured of the confidentiality and anonymity of their data. The HRS is sponsored by the National Institute on Aging (grant number NIA U01AG009740) to the University of Michigan. As data were publicly available, IRB approval was not required for this analysis.

### Participant selection

**Fig 1:**
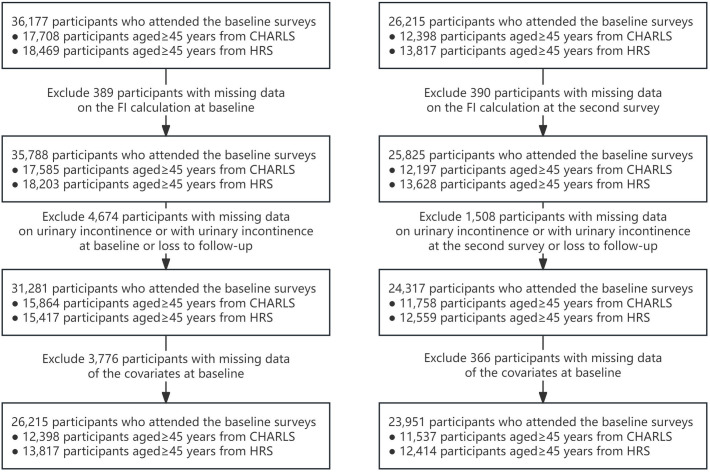
Flowchart of sample selection. Selection process of the study population. CHARLS, China Health and Retirement Longitudinal Study; HRS, Health and Retirement Study; FI, frailty index.

Fig 1 shows the selection process of the study population. From a total of 36,177 individuals in both studies, we carefully selected our study population. We first removed 389 people who lacked information needed to calculate their frailty index at the start. We also excluded 4,674 people with missing data on UI or with UI at the beginning of the study or loss to follow-up. Additionally, 3,776 people with missing information on other important factors were excluded. This left us with 26,215 eligible participants for our initial analyses of frailty. For analyses focusing on changes in frailty over time, we further excluded 2,264 participants by their two-year follow-up data based on similar criteria, resulting in a final group of 23,951 participants. A comparison with excluded participants revealed significant baseline differences (S1 Table), with excluded individuals generally being older and having more comorbidities. To mitigate this potential selection bias, our analytical models were adjusted for these key demographic and health variables.

### Measuring frailty

Frailty was measured using a frailty index (FI), which is a score based on the number of health problems an individual has [9,10,14]. We used a standard method to create this index, including 30 different health indicators from the CHARLS and HRS data (see S2 Table). These indicators covered various aspects like diseases, symptoms, disabilities, physical and cognitive function, mental health, and functional limitations as measured by Activities of Daily Living (ADLs) and Instrumental Activities of Daily Living (IADLs). For most indicators, a score of 1 meant the problem was present, and 0 meant it was absent. One item, a cognitive score, was a continuous value from 0 to 1 where a higher score indicated worse thinking ability. The FI for each person was calculated by adding up all their present health problems and dividing by 30. This gave a score between 0 and 1, a higher score meant a higher level of frailty. Based on previous research, we categorized frailty into three groups: Robust: FI of 0.10 or less; Pre-frail: FI between 0.10 and 0.25; Frail: FI of 0.25 or more [6,15,16]. We also looked at changes in frailty by comparing the FI at the start of the study with the FI two years later. We calculated change in FI (2nd survey FI minus baseline FI) to understand the dynamic nature of frailty.

### Other factors considered

We included several other factors in our analyses: age, sex, marital status, education level, residence, smoking habits, drinking habits, body mass index (BMI), hypertension, and diabetes. To ensure consistency between the CHARLS and HRS data, marital status was grouped into “married or partnered” and “other” (separated, divorced, unmarried, or widowed). Education was classified as “below high school,” “high school,” and “college or above.” Given their established roles as potential risk factors for UI, smoking and drinking habits were included as important confounders [17,18]. Smoking and drinking habits were categorized as “never” or “ever” users.

### Outcome and follow-up

The primary outcome was new cases of UI. In both studies, UI was identified by asking participants if they had difficulty controlling urination. For analyses focusing on initial frailty status, follow-up began at the first survey (Wave 1 of CHARLS, Wave 8 of HRS). For analyses looking at changes in frailty, follow-up began at the second survey (Wave 2 of CHARLS, Wave 9 of HRS). Participants were followed until they either developed UI, passed away, or reached the last survey they attended, whichever came first. Ideally, this last survey was in 2020. Information on deaths was available up to the final survey in both studies.

### Statistical analyses

For basic descriptions of the data, we used means and standard deviations for numerical data, and counts and percentages for categories.To examine the link between initial frailty status and the risk of developing UI, we used Cox regression to calculate hazard ratios (HRs) and their 95% confidence intervals (CIs). Cox regression model was adjusted for age, sex, marital status, education, residence, smoking, drinking, high blood pressure, and diabetes. We used individuals categorized as “robust” as the comparison group. We used similar methods to analyze how changes in frailty, total FI, and change in FI related to the development of UI. We also conducted subgroup analyses based on age, sex, marital status, education, residence, smoking, drinking, high blood pressure, and diabetes to see if the relationships differed in these groups.

All statistical calculations were performed using R software (Version 4.3.1). A p-value less than 0.05 was considered statistically significant.

## Results

### Baseline characteristics of the study participants

After applying our selection criteria, we included 12,398 participants from CHARLS (52.3% female, average age 59.3 years) and 13,817 participants from HRS (53.3% female, average age 67.3 years) for the analyses of baseline frailty. As shown in Table 1, participants classified as frail in both studies were generally older, more often female, less likely to be married or partnered, and had lower levels of education compared to those who were robust. Additionally, frail participants had higher rates of hypertension and diabetes than robust participants. For the analyses focusing on changes in frailty status, we included 11,535 participants from CHARLS (52.7% female, average age 60.3 years) and 12,559 from HRS (53.1% female, average age 68.5 years), based on similar inclusion criteria. The characteristics of these participants at baseline are presented in Table 2.

**Table 1:** Baseline characteristics of participants for baseline frailty status analyses.

	CHARLS(*N = 12,398*)				HRS(*N = 13,817*)			
Variable	robust, N = 4,019	pre-frailty, N = 4,897	frailty, N = 3,482	p-value	robust, N = 3,999	pre-frailty, N = 4,393	frailty, N = 5,425	p-value
**Age (years), Mean (SD)**	56.2 (8.5)	59.3 (9.2)	63.0 (9.8)	<0.001	61.7 (8.8)	67.6 (9.5)	71.2 (10.6)	<0.001
**Gender, n (%)**				<0.001				<0.001
Female	1,767 (44%)	2,499 (51%)	2,222 (64%)		2,045 (51%)	2,187 (50%)	3,136 (58%)	
Male	2,252 (56%)	2,398 (49%)	1,260 (36%)		1,954 (49%)	2,206 (50%)	2,289 (42%)	
**Marital status, n (%)**				<0.001				<0.001
Married or partnered	3,504 (87%)	4,068 (83%)	2,663 (76%)		2,920 (73%)	2,943 (67%)	2,890 (53%)	
Other	515 (13%)	829 (17%)	819 (24%)		1,079 (27%)	1,450 (33%)	2,535 (47%)	
**Education level, n (%)**				<0.001				<0.001
Below high school	3,347 (83%)	4,431 (90%)	3,293 (95%)		444 (11%)	850 (19%)	1,788 (33%)	
High school	574 (14%)	401 (8%)	166 (5%)		2,180 (55%)	2,638 (60%)	2,977 (55%)	
College or above	98 (2%)	65 (1%)	23 (1%)		1,375 (34%)	905 (21%)	660 (12%)	
**Residence**				<0.001				<0.001
Urban	1,642 (41%)	1,900 (39%)	1,130 (32%)		2,873 (72%)	2,999 (68%)	3,589 (66%)	
Rural	2,377 (59%)	2,997 (61%)	2,352 (68%)		1,126 (28%)	1,394 (32%)	1,836 (34%)	
**Smoking status, n (%)**				<0.001				<0.001
Never smokers	2,272 (57%)	2,895 (59%)	2,274 (65%)		1,910 (48%)	1,842 (42%)	2,097 (39%)	
Ever smokers	1,747 (43%)	2,002 (41%)	1,208 (35%)		2,089 (52%)	2,551 (58%)	3,328 (61%)	
**Drinking status, n (%)**				<0.001				<0.001
Never drinkers	2,475 (62%)	3,190 (65%)	2,631 (76%)		1,408 (35%)	1,970 (45%)	3,333 (61%)	
Ever drinkers	1,544 (38%)	1,707 (35%)	851 (24%)		2,591 (65%)	2,423 (55%)	2,092 (39%)	
**Body mass index (kg/m²), Mean (SD)**	24.8 (52.1)	24.2 (36.3)	23.7 (4.4)	<0.001	26.5 (4.4)	27.6 (5.1)	28.6 (6.3)	<0.001
**Hypertension, n (%)**				<0.001				<0.001
No	3,728 (93%)	3,478 (71%)	2,022 (58%)		3,018 (75%)	1,926 (44%)	1,522 (28%)	
Yes	291 (7%)	1,419 (29%)	1,460 (42%)		981 (25%)	2,467 (56%)	3,903 (72%)	
**Diabetes, n (%)**				<0.001				<0.001
No	3,970 (99%)	4,616 (94%)	3,067 (88%)		3,861 (97%)	3,723 (85%)	3,726 (69%)	
Yes	49 (1%)	281 (6%)	415 (12%)		138 (3%)	670 (15%)	1,699 (31%)	

CHARLS, China Health and Retirement Longitudinal Study; HRS, Health and Retirement Study; BMI, body mass index.

**Table 2:** Baseline characteristics of participants for changes in frailty status analyses.

	CHARLS(*N = 11,537*)				HRS(*N = 12,414*)			
Variable	robust, N = 3,411	pre-frailty, N = 4,695	frailty, N = 3,431	p-value	robust, N = 3,342	pre-frailty, N = 4,102	frailty, N = 4,970	p-value
**Age (years), Mean (SD)**	57.9 (9.1)	60.0 (9.2)	63.2 (9.7)	<0.001	62.9 (8.6)	68.6 (9.5)	72.1 (10.1)	<0.001
**Gender, n (%)**				<0.001				<0.001
Female	1,473 (43%)	2,383 (51%)	2,225 (65%)		1,726 (52%)	2,057 (50%)	2,803 (56%)	
Male	1,938 (57%)	2,312 (49%)	1,206 (35%)		1,616 (48%)	2,045 (50%)	2,167 (44%)	
**Marital status, n (%)**				<0.001				<0.001
Married or partnered	2,948 (86%)	3,953 (84%)	2,655 (77%)		2,396 (72%)	2,679 (65%)	2,650 (53%)	
Other	463 (14%)	742 (16%)	776 (23%)		946 (28%)	1,423 (35%)	2,320 (47%)	
**Education level, n (%)**				<0.001				<0.001
Below high school	2,889 (85%)	4,181 (89%)	3,215 (94%)		335 (10%)	737 (18%)	1,598 (32%)	
High school	440 (13%)	451 (10%)	185 (5%)		1,825 (55%)	2,440 (59%)	2,794 (56%)	
College or above	82 (2%)	63 (1%)	31 (1%)		1,182 (35%)	925 (23%)	578 (12%)	
**Residence**				<0.05				<0.001
Urban	1,287 (38%)	1,741 (37%)	1,199 (35%)		2,417 (72%)	2,807 (68%)	3,289 (66%)	
Rural	2,124 (62%)	2,954 (63%)	2,232 (65%)		925 (28%)	1,295 (32%)	1,681 (34%)	
**Smoking status, n (%)**				<0.001				<0.001
Never smokers	1,801 (53%)	2,668 (57%)	2,213 (65%)		1,613 (48%)	1,706 (42%)	1,915 (39%)	
Ever smokers	1,610 (47%)	2,027 (43%)	1,218 (35%)		1,729 (52%)	2,396 (58%)	3,055 (61%)	
**Drinking status, n (%)**				<0.001				<0.001
Never drinkers	2,000 (59%)	2,999 (64%)	2,611 (76%)		1,175 (35%)	1,831 (45%)	3,088 (62%)	
Ever drinkers	1,411 (41%)	1,696 (36%)	820 (24%)		2,167 (65%)	2,271 (55%)	1,882 (38%)	
**Body mass index (kg/m²), Mean (SD)**	39.2 (902.6)	23.9 (5.5)	24.5 (7.6)	<0.001	26.5 (4.5)	27.7 (5.1)	28.7 (6.5)	<0.001
**Hypertension, n (%)**				<0.001				<0.001
No	3,154 (92%)	3,278 (70%)	1,873 (55%)		2,477 (74%)	1,693 (41%)	1,225 (25%)	
Yes	257 (8%)	1,417 (30%)	1,558 (45%)		865 (26%)	2,409 (59%)	3,745 (75%)	
**Diabetes, n (%)**				<0.001				<0.001
No	3,364 (99%)	4,377 (93%)	2,963 (86%)		3,202 (96%)	3,386 (83%)	3,341 (67%)	
Yes	47 (1%)	318 (7%)	468 (14%)		140 (4%)	716 (17%)	1,629 (33%)	

CHARLS, China Health and Retirement Longitudinal Study; HRS, Health and Retirement Study; BMI, body mass index.

### Association of baseline frailty status with new cases of Urinary Incontinence

As shown in Table 3, we found a clear link between a person’s frailty status at the start of the study and their risk of developing UI. Even after accounting for other influencing factors, frail participants in both studies had a significantly higher risk of developing UI compared to robust participants. Specifically, in the CHARLS study, frail individuals were 4.51 times more likely to develop UI (95% CI: 3.77–5.38), while in the HRS study, they were 2.25 times more likely (95% CI: 2.07–2.46). Similarly, pre-frail participants also showed a significantly increased risk of developing UI compared to robust participants. In CHARLS, pre-frail individuals had a 2.14 times higher risk (95% CI: 1.79–2.55), and in HRS, their risk was 1.50 times higher (95% CI: 1.38–1.62).

**Table 3:** Association of frailty status with risks of Urinary Incontinence.

	CHARLS			HRS		
Frailty Status	%	HR (95% CI)	P-value	%	HR (95% CI)	P-value
***robust***	*32.5%*	*Reference*		*30.2%*	*Reference*	
**pre-frailty**	39.9%	2.14 (1.79-2.55)	<0.001	32.7%	1.50 (1.38-1.62)	<0.001
**frailty**	27.6%	4.51 (3.77-5.38)	<0.001	37.1%	2.25 (2.07-2.46)	<0.001

HR: Hazard Ratio; CI: Confidence Interval.

Model adjusted for age, residence, marital status, education, smoking, drinking, BMI, hypertension, diabetes.

### Association of Changes in Frailty Status with New Cases of Urinary Incontinence

Table 4 shows how changes in frailty status over time relate to the risk of developing UI. Compared to participants who remained robust, those who were robust initially but then became pre-frail or frail had a significantly higher risk of developing UI. Specifically, in CHARLS, their risk was 2.81 times higher (95% CI: 1.94–4.08), and in HRS, it was 1.44 times higher (95% CI: 1.24–1.68). Conversely, we observed a significant decrease in UI risk among frail participants who improved to a robust or pre-frail status, when compared to those who remained frail. In CHARLS, their risk was 0.51 times lower (95% CI: 0.41–0.62), and in HRS, it was 0.79 times lower (95% CI: 0.69–0.90). For participants who were pre-frail at the start, those who then progressed to being frail showed a significantly increased risk of UI compared to those who stayed pre-frail. This risk was 1.65 times higher in CHARLS (95% CI: 1.28–2.12) and 1.16 times higher in HRS (95% CI: 1.03–1.32). While pre-frail participants who improved to robust status also had a decreased risk of UI, this reduction was not statistically significant in either study (CHARLS: HR 0.88, 95% CI: 0.64–1.21; HRS: HR 0.93, 95% CI: 0.78–1.11). In summary, individuals who became more frail had a higher risk of developing UI, while those who recovered from frailty saw a reduced risk.

**Table 4:** Association of changes in frailty status with risks of Urinary Incontinence.

		CHARLS			HRS		
Frailty Status	Change	%	HR (95% CI)	P-value	%	HR (95% CI)	P-value
**robust**	*no change*	*19.7%*	*1.00 (Reference)*		*24.1%*	*1.00 (Reference)*	
	worsened	15.2%	2.81 (1.94-4.08)	<0.001	8.2%	1.44 (1.24-1.68)	<0.001
**pre-frailty**	*no change*	*21.5%*	*1.00 (Reference)*		*21.5%*	*1.00 (Reference)*	
	improved	7.8%	0.88 (0.64-1.21)	0.434	3.8%	0.93 (0.78-1.11)	0.424
	worsened	9.7%	1.65 (1.28-2.12)	<0.001	8.4%	1.16 (1.03-1.32)	<0.05
**frailty**	*no change*	*17.1%*	*1.00 (Reference)*		*28.2%*	*1.00 (Reference)*	
	improved	9.0%	0.51 (0.41-0.62)	<0.001	5.7%	0.79 (0.69-0.90)	<0.001

HR: Hazard Ratio; CI: Confidence Interval.

Model adjusted for age, residence, marital status, education, smoking, drinking, BMI, hypertension, diabetes.

### Subgroup analysis

**Fig 2:**
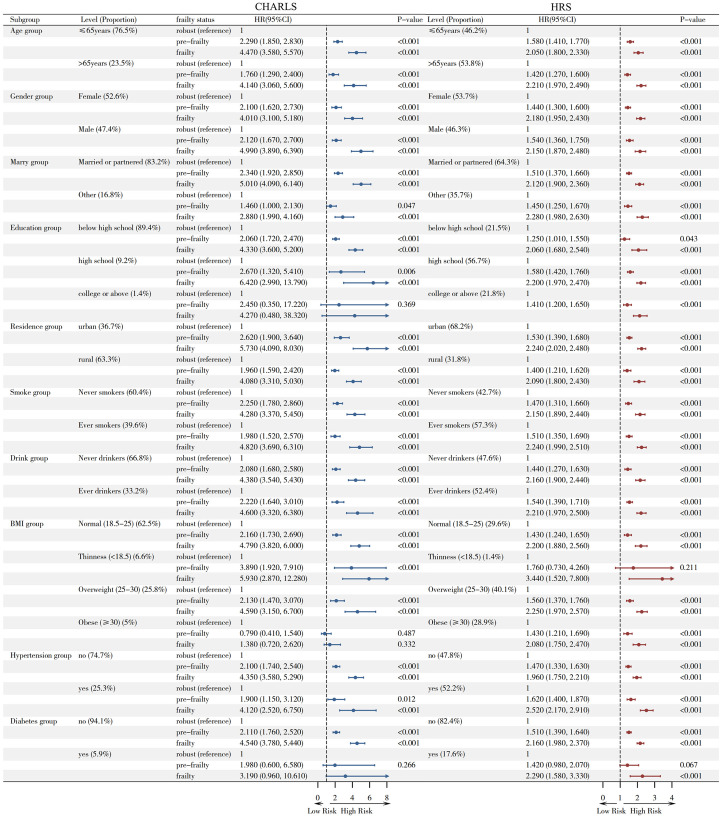
Subgroup analyses for the association of frailty status with risks of Urinary Incontinence by potential mediators. HR: Hazard Ratio; CI: Confidence Interval; Frailty categorized as improved/no change/worsened with no change as reference; Model adjusted for: age, gender, marital status, education level, residence area, smoking status, drinking status, BMI, hypertension, diabetes.

Fig 2 shows the subgroup analyses of the association between frailty status and incident UI, by age, gender, marital status, education level, residence area, smoking status, drinking status, BMI, hypertension, diabetes. Similar results were observed across all subgroups.

## Discussion

The primary findings of our study indicate that older adults identified as pre-frail and frail had a significantly increased hazard ratio (HR) for developing urinary incontinence(UI) compared to individuals classified as robust at baseline. Furthermore, we observed a clear dose-response relationship, where the HR for UI progressively increased with rising frailty severity, from pre-frailty to frailty, indicating that the risk of UI increases with the degree of frailty. When examining the impact of changes in frailty status over time, our analysis indicated that among individuals who were robust or pre-frail at baseline, those whose frailty status worsened during follow-up had a significantly increased HR for developing UI compared to those with no change in frailty status. Conversely, our findings showed that among individuals who were frail at baseline, the recovery of frailty status over time was associated with a reduced HR for UI compared to the group with no change in frailty status. Similar results were observed across subgroups based on a range of demographic and health-related covariates.

Our findings are consistent with and expand upon existing research that has established a significant association between frailty and UI in older adults. The increased risk of UI observed in frail individuals in our study is consistent with the results of the meta-analysis by Lee et al., which reported a pooled odds ratio of 2.28 for UI in frail older adults compared to non-frail older adults [11]. Our longitudinal findings extend the evidence from previous longitudinal studies, such as those by Miles et al. and Chong et al., which suggested that incident UI may serve as an early indicator of frailty and that frailty predicts the occurrence of UI [19,20]. Our study adds further depth by demonstrating the impact of changes in frailty status over time on UI risk. While previous studies have established a cross-sectional association between frailty and UI, our study provides novel and compelling evidence regarding the longitudinal impact of baseline frailty and subsequent changes in frailty status on UI risk. Utilizing large, nationally representative data from China and the United States enhances the robustness and generalizability of these findings, placing them within the context of global health challenges posed by aging populations and related public health strategies like the “Healthy China 2030” initiative [21].

Several interconnected biological mechanisms may contribute to the observed association between frailty and UI [19]. Age-related loss of skeletal muscle mass and strength, known as sarcopenia, is a core component of the frailty syndrome [5]. This decline in muscle function can directly affect the strength and coordination of the pelvic floor muscles, which play a crucial role in maintaining urinary continence, thereby increasing the risk of UI [22]. Furthermore, sarcopenia is often associated with reduced overall physical activity and mobility, which can indirectly impact continence mechanism [23]. The close association between sarcopenia and UI suggests that interventions aimed at preserving or improving muscle mass and strength, such as targeted exercise programs and adequate nutritional support, may contribute to reducing the risk of UI in frail older adults. Age-related changes in the nervous system, as well as neurological conditions more prevalent in frail older adults, such as stroke, dementia, and Parkinson’s disease, can impair bladder control and contribute to the development of UI [24]. The complex brain-bladder axis, responsible for regulating the micturition reflex, can be influenced by the aging process and neurological damage, resulting in reduced precision of central nervous system control over bladder function [25]. The involvement of neural factors highlights the complex interplay between frailty and UI, suggesting that the cognitive and functional declines often associated with frailty can indirectly impact an individual’s ability to recognize and appropriately respond to bladder signals, thus increasing the likelihood of UI. Chronic low-grade inflammation is increasingly recognized as a significant contributor to the pathogenesis of frailty [5]. Elevated pro-inflammatory cytokines can negatively impact muscle function, disrupt neural pathways involved in bladder control, and potentially directly affect bladder function, thereby contributing to the development of frailty and increased UI risk [26]. The potential role of chronic inflammation in the frailty-UI link suggests that interventions targeting inflammatory pathways, such as lifestyle modifications and potential pharmacological approaches, may be beneficial for both conditions. It is likely that these and other potential biological mechanisms collectively contribute in complex and interacting ways to the multifaceted relationship between frailty and UI in older adults. Further research is needed to fully elucidate these underlying pathways and their relative contributions.

The strong association between improving frailty and reducing UI risk highlights the importance of proactive interventions. Evidence suggests that multicomponent interventions are most effective for managing frailty [27]. These programs typically combine physical exercise (including resistance training to combat sarcopenia, balance training, and specific Pelvic Floor Muscle Training), nutritional support (e.g., ensuring adequate protein and vitamin D intake), and cognitive stimulation [28]. By addressing the root causes of frailty, such programs hold significant promise for preventing or delaying the onset of UI in vulnerable older adults.

A key strength of our study is the utilization of large, nationally representative longitudinal datasets, namely the China Health and Retirement Longitudinal Study (CHARLS) and the Health and Retirement Study (HRS), which significantly enhances the generalizability of our findings to broader populations. The use of a comprehensive Frailty Index comprising 30 variables allowed for a detailed and comprehensive assessment of frailty status, capturing a wide range of age-related deficits. Our longitudinal analysis approach enabled us to examine the temporal relationship between baseline frailty levels and subsequent changes in frailty status and the risk of incident UI, providing stronger evidence for the direction of this association compared to cross-sectional studies. The inclusion of a range of relevant covariates in our Cox regression models helped to control for potential confounding factors that could influence the observed association. Despite its strengths, several limitations of this study should be considered when interpreting the findings. The 30-variable Frailty Index, while comprehensive, represents only one operational definition of frailty; other indices or phenotypic definitions might yield different frailty classifications and potentially different associations with UI [8]. The specific selection of variables included in the index might influence the identification of frail individuals. Our assessment of UI relied on self-reported data, which is susceptible to recall bias and potential underreporting due to factors such as embarrassment, social stigma, or the perception that UI is a normal part of aging [29]. Due to limitations in the datasets, we were unable to adjust for the use of specific medications known to affect urinary function (e.g., diuretics, SGLT2 inhibitors, psychoactive drugs), which could be a source of residual confounding. The observational nature of our study precludes us from establishing a definitive causal relationship between frailty and UI. While our longitudinal design provides strong evidence that frailty precedes UI, we cannot entirely rule out a bidirectional or “vicious cycle” relationship where UI might, in turn, exacerbate frailty.

## Conclusion

In summary, our study provides compelling evidence for a significant association between baseline frailty and changes in frailty status with the risk of Urinary Incontinence in older adults. Individuals exhibiting higher levels of frailty at baseline faced a greater risk of developing Urinary Incontinence, and the progression of frailty status over time further enlarged this risk, while conversely, recovery of frailty was associated with a decreased risk. Future research should prioritize investigating the underlying mechanisms contributing to this association and developing targeted interventions aimed at addressing frailty to effectively prevent or manage this common and often debilitating condition.

## Supporting information

S1 Table Comparison of baseline characteristics between included and excluded participants. (DOCX)

S2 Table The 30 items used to construct the frailty index. (DOCX)
