# Correction: Impact of frailty and its change on urinary incontinence: A longitudinal analysis from two prospective study of ageing

**DOI:** 10.1371/journal.pone.0342254

**Published:** 2026-02-03

**Authors:** Xiaohan Gan, Linghao Meng, Shangqi Cao, Hexiang Bai, Xiang Li

The Funding statement for this article is incorrect. The correct Funding statement is as follows: This work was financially supported by the Chengdu Science and Technology Program (2024-YF05–00216-SN), received by Xiang Li. The funder had no role in study design, data collection and analysis, decision to publish, or preparation of the manuscript.

## References

[1] Gan X, Meng L, Cao S, Bai H, Li X. Impact of frailty and its change on urinary incontinence: A longitudinal analysis from two prospective study of ageing. PLoS One. 2025;20(8): e0330062. doi: 10.1371/journal.pone.0330062 PMC12367120 40833979

