# Supplementary material for: Impact of frailty and its change on urinary incontinence: A longitudinal analysis from two prospective study of ageing
Source: PLoS One. 2025 Aug 20;20(8):e0330062. doi: 10.1371/journal.pone.0330062 (PMC12367120; doi:10.1371/journal.pone.0330062)
Supplement: S1 Table — (DOCX) [file pone.0330062.s001.docx]

**S1 Table.** Comparison of Baseline Characteristics Between Included and Excluded Participants

|  | **CHARLS** | | | **HRS** | | |
| --- | --- | --- | --- | --- | --- | --- |
| **Variable** | **Deleted**, N = 5,310^1^ | **Included**, N = 12,398^1^ | **p-value** | **Deleted**, N = 4,652^1^ | **Included**, N = 13,817^1^ | **p-value** |
| **Frailty status** |  |  | <0.001 |  |  | <0.001 |
| Robust | 2,157 (42%) | 4,019 (32%) |  | 626 (13%) | 3,999 (29%) |  |
| Pre-frailty | 1,410 (27%) | 4,897 (39%) |  | 1,091 (23%) | 4,393 (32%) |  |
| Frailty | 1,620 (31%) | 3,482 (28%) |  | 2,935 (63%) | 5,425 (39%) |  |
| **Age (years)** **, Mean (SD)** | 58 (12) | 59 (10) | <0.001 | 70 (13) | 67 (11) | <0.001 |
| **Gender, n (%)** |  |  | 0.4 |  |  | <0.001 |
| Female | 2,739 (52%) | 6,488 (52%) |  | 3,515 (76%) | 7,368 (53%) |  |
| Male | 2,569 (48%) | 5,910 (48%) |  | 1,137 (24%) | 6,449 (47%) |  |
| **Marital status, n (%)** |  |  | <0.001 |  |  | <0.001 |
| Married or partnered | 3,935 (75%) | 10,235 (83%) |  | 2,520 (54%) | 8,753 (63%) |  |
| Other | 1,342 (25%) | 2,163 (17%) |  | 2,132 (46%) | 5,064 (37%) |  |
| **Education level, n (%)** |  |  | <0.001 |  |  | <0.001 |
| Below high school | 4,454 (84%) | 11,071 (89%) |  | 1,070 (23%) | 3,082 (22%) |  |
| High school | 667 (13%) | 1,141 (9.2%) |  | 2,723 (59%) | 7,795 (56%) |  |
| College or above | 174 (3.3%) | 186 (1.5%) |  | 855 (18%) | 2,940 (21%) |  |
| **Residence** |  |  | <0.001 |  |  | 0.7 |
| Urban | 2,499 (47%) | 4,672 (38%) |  | 3,143 (69%) | 9,461 (68%) |  |
| Rural | 2,811 (53%) | 7,726 (62%) |  | 1,424 (31%) | 4,356 (32%) |  |
| **Smoking status, n (%)** |  |  | 0.034 |  |  | 0.010 |
| Never smokers | 3,187 (62%) | 7,441 (60%) |  | 2,002 (45%) | 5,849 (42%) |  |
| Ever smokers | 1,974 (38%) | 4,957 (40%) |  | 2,494 (55%) | 7,968 (58%) |  |
| **Drinking status, n (%)** |  |  | 0.3 |  |  | <0.001 |
| Never drinkers | 3,489 (68%) | 8,296 (67%) |  | 2,583 (56%) | 6,711 (49%) |  |
| Ever drinkers | 1,665 (32%) | 4,102 (33%) |  | 2,062 (44%) | 7,106 (51%) |  |
| **Body mass index (kg/m²), Mean (SD)** | 23.4 (3.8) | 24.2 (37.5) | <0.05 | 28.4 (6.8) | 27.7 (5.5) | <0.001 |
| **Hypertension, n (%)** |  |  | 0.069 |  |  | <0.001 |
| No | 3,707 (73%) | 9,228 (74%) |  | 1,888 (41%) | 6,466 (47%) |  |
| Yes | 1,365 (27%) | 3,170 (26%) |  | 2,764 (59%) | 7,351 (53%) |  |
| **Diabetes, n (%)** |  |  | 0.4 |  |  | <0.001 |
| No | 4,693 (94%) | 11,653 (94%) |  | 3,624 (78%) | 11,310 (82%) |  |
| Yes | 317 (6.3%) | 745 (6.0%) |  | 1,028 (22%) | 2,507 (18%) |  |

CHARLS, China Health and Retirement Longitudinal Study; HRS, Health and Retirement Study; BMI, body mass index.
