# Supplementary material for: Impact of frailty and its change on urinary incontinence: A longitudinal analysis from two prospective study of ageing
Source: PLoS One. 2025 Aug 20;20(8):e0330062. doi: 10.1371/journal.pone.0330062 (PMC12367120; doi:10.1371/journal.pone.0330062)
Supplement: S2 Table — (DOCX) [file pone.0330062.s002.docx]

| **S2 Table**. The 30 items used to construct the frailty index | | | |  |
| --- | --- | --- | --- | --- |
| No | Description of the item | | Cut-off value |  |
|  | CHARLS | HRS |  |  |
| 1 | Self-reported physician diagnosed hypertension | | Yes = 1, No = 0 |  |
| 2 | Self-reported physician diagnosed diabetes | | Yes = 1, No = 0 |  |
| 3 | Self-reported physician diagnosed cancer | | Yes = 1, No = 0 |  |
| 4 | Self-reported physician diagnosed arthritis | | Yes = 1, No = 0 |  |
| 5 | Self-reported physician diagnosed chronic lung disease | | Yes = 1, No = 0 |  |
| 6 | Self-reported physician diagnosed heart disease | | Yes = 1, No = 0 |  |
| 7 | Self-reported physician diagnosed stroke | | Yes = 1, No = 0 |  |
| 8 | Self-reported physician diagnosed any emotional, nervous, or psychiatric problems | | Yes = 1, No = 0 |  |
| 9 | Self-reported physician diagnosed memory-related disease | | Yes = 1, No = 0 |  |
| 10 | Self-reported vision problems | Self-reported eyesight (while using lenses if appropriate) | Yes = 1, No = 0 in the CHARLS; Poor or fair = 1, excellent, very good, or good = 0 in the HRS |  |
| 11 | Self-reported hearing problems | Self-reported hearing (while using hearing aid if appropriate) | Yes = 1, No = 0 in the CHARLS; Poor or fair = 1, excellent, very good, or good = 0 in the HRS |  |
|  |  |  |  |  |
| 12 | Self-reported general health status | | Very poor or poor = 1, Very good, good, or fair = 0 |  |
| 13 | Difficulty with dressing | | Yes = 1, No = 0 |  |
| 14 | Difficulty with bathing or showering | | Yes = 1, No = 0 |  |
| 15 | Difficulty with eating | | Yes = 1, No = 0 |  |
| 16 | Difficulty with getting in and out of bed | | Yes = 1, No = 0 |  |
| 17 | Difficulty with using the toilet | | Yes = 1, No = 0 |  |
| 18 | Difficulty with managing money | | Yes = 1, No = 0 |  |
| 19 | Difficulty with taking medication | | Yes = 1, No = 0 |  |
| 20 | Difficulty with shopping for groceries | | Yes = 1, No = 0 |  |
| 21 | Difficulty with preparing meals | | Yes = 1, No = 0 |  |
| 22 | Mobility: difficulty with walking 100 yards or one block | | Yes = 1, No = 0 |  |
| 23 | Mobility: difficulty with getting up from a chair after sitting for long periods | | Yes = 1, No = 0 |  |
| 24 | Mobility: difficulty with climbing several flights of stairs without resting | | Yes = 1, No = 0 |  |
| 25 | Mobility: difficulty with lifting or carrying weights over 10 pounds/jins | | Yes = 1, No = 0 |  |
| 26 | Mobility: difficulty with picking up a coin from the table | | Yes = 1, No = 0 |  |
| 27 | Mobility: difficulty with stooping, kneeling, or crouching | | Yes = 1, No = 0 |  |
| 28 | Mobility: difficulty with reaching arms above shoulder level | | Yes = 1, No = 0 |  |
| 29 | Depression: CESD-10 questionnaire | Depression: CESD-8 questionnaire | CESD-10 >10 = 1, ≤10 = 0 in the CHARLS; CESD-8 ≥ 4 = 1, <4 = 0 in the HRS |  |
| 30 | Cognition: (memory test score + orientation test score) **/** 14 | | Continuous variable, ranging from 0 to 1 |  |
| Memory-related disease included Alzheimer’s disease, dementia, organic brain senility, and other serious memory impairment.  Depression was assessed by the Center for Epidemiologic Studies Depression Scale (CESD). In the CHARLS, CESD-10 was used, and the total score ranged from 0 to 30. In the HRS, CESD-8 was used, and the total score ranged from 0 to 8. A higher score indicated more severe depressive symptoms.  The memory score was the average of words which were not recalled in the immediate and delayed word recall tasks. The memory score ranged from 0 to 10. The orientation test comprised 4 questions about the day of the week, the month, the date of the month, and the year. One point was given for each wrong answer, and the range was from 0 to 4. | | | |  |
| CHARLS, China Health and Retirement Longitudinal Study; HRS, Health and Retirement Study | | | |  |
